# Surgical experience of adult primary hepatic sarcomas

**DOI:** 10.1186/s12957-015-0489-6

**Published:** 2015-02-28

**Authors:** Yu-Hung Lin, Chih-Che Lin, Allan M Concejero, Chee-Chien Yong, Fang-Ying Kuo, Chih-Chi Wang

**Affiliations:** Department of Surgery of Kaohsiung Chang Gung Memorial Hospital, Chang Gung University College of Medicine, No. 123, Da-Pei Road, Niao-Sung, Kaohsiung 83301 Taiwan; Department of Pathology of Kaohsiung Chang Gung Memorial Hospital, Chang Gung University College of Medicine, Kaohsiung, 83301 Taiwan

**Keywords:** Primary hepatic sarcoma, Surgery, Angiosarcoma, Non-angiosarcoma, Outcome

## Abstract

**Background:**

Primary hepatic sarcoma (PHS) is a rare primary liver malignancy. The histological types of PHS are diverse, and the clinical outcomes and management mainly depend on the histopathology. This study aims to evaluate the results of surgical intervention.

**Methods:**

Between January 2003 and June 2009, 13 adult patients with pathologically proven PHS were identified by record review. The patients’ demographic profile, tumor characteristics, treatment modalities, and outcomes were reviewed and analyzed. The end of follow-up was December 2014.

**Results:**

Nine (69%) underwent curative liver resection and two underwent liver transplantation; the others received non-operative treatments. The pathologic findings were six (46%) angiosarcomas, four (30.7%) undifferentiated sarcomas, one (7.6%) leiomyosarcoma, one (7.6%) malignant mesenchymoma, and one (7.6%) hepatic epithelioid hemangioendothelioma. The median follow-up was 31.4 (2.8 ~ 142.5) months. The 1-, 2-, and 5-year survival of surgical patients were 72.7%, 63.6%, and 36.4%, respectively. Importantly, the 1-, 2-, and 5-year survival rates of non-angiosarcoma patients were superior to those of angiosarcoma (85.7% vs. 33.3%, 71.4% vs. 16.7%, and 57.1% vs. 0%, respectively, *P* = 0.023).

**Conclusions:**

Surgical intervention provides the possibility of long-term survival from PHS. Angiosarcoma is associated with a more dismal outcome than non-angiosarcoma.

## Background

Primary hepatic sarcoma (PHS) is a rare malignancy accounting for less than 1% of all liver malignancies [1-3]. The etiologies of this disease are still not well-known, unlike hepatocellular carcinoma (HCC) which is the most common histology of primary hepatic malignancy and closely associated with infection of viral hepatitis, alcoholism, and liver cirrhosis. The clinical presentations of PHS are usually non-specific such as abdominal pain and weight loss [4,5]. HCC and cholangiocarcinoma are associated with the elevation of alpha-fetoprotein (AFP) and carbohydrate antigen 19-9 (CA 19-9), respectively. But no tumor marker has been identified to accurately detect PHS. The treatment guidelines of HCC evolved and have been very well-established in recent decades [4]. In contrast, PHS is less studied and no clinical guideline can be applied.

PHS has a wide diversity of histological types. Leiomyosarcoma and angiosarcoma are the most common histological types [1,6-9]. Undifferentiated sarcoma most often occurs in childhood but is also common in adults. The histological nomenclature for undifferentiated sarcoma was not uniform before 1978. The names were mesenchymoma, primary sarcoma of liver, fibromyxosarcoma, and malignant mesenchymoma.

The clinical outcomes and treatment are quite different in each type of histology. Radiotherapy is commonly used for extremity sarcoma, but its detrimental adverse effects when delivered at high doses to abdominal organs limit its application when treating hepatic sarcoma [1]. Without surgery, chemotherapy provides minimal benefits of survival in sarcomas. In the past two decades, substantial improvement in surgical technique, perioperative management, and earlier diagnosis have resulted in marked reduction in operative and hospital mortality rates for patients undergoing hepatectomy and liver transplantation (LT) [10-13]. Aggressive surgical approach seems to be the only effective treatment to achieve possible long-term survival for PHS [7].

Since PHS is rare, the course of disease, prognostic factors, and consensus on management are not well-clarified. The aim of this study is to analyze our surgical experience in the management of this rare malignancy.

## Methods

We retrospectively reviewed the records of 13 adult patients with PHS confirmed by pathology at Kaohsiung Chang Gung Memorial Hospital from January 2003 to June 2009. Patients were followed up till December 2014. This study was approved by the Institutional Review Board of this hospital. Written informed consent was obtained from the patient for the publication of this report and any accompanying images.

The diagnostic imaging modalities were abdominal ultrasound, computed tomography (CT), or magnetic resonance imaging (MRI) with or without hepatic angiography. Laboratory blood tests included hepatitis B antigen and antibody, hepatitis C antibody, serum AFP, carcinoembryonic antigen (CEA), CA 19-9, serum albumin, serum total bilirubin, aspartate aminotransferase, alanine aminotransferase, differentiated blood cell counts, international normalized ratio, and prothrombin time.

Patients who underwent work-up for possible LT underwent other examinations that included panendoscopy, colonoscopy, chest and brain images, and bone scan to screen out possible distant metastases or double cancers. Patients who were identified as candidates for hepatic resection underwent indocyanine green clearance test [10,11]. Resectability was determined by tumor extent, possible residual liver volume on imaging, and biochemical tests especially indocyanine green clearance test [14]. Patients with resectable liver tumors and had resectable solitary lung metastasis were also considered as resection candidates.

The surgery types were hepatectomy (anatomical and non-anatomical) and LT (brain-death deceased donor and living-related donor). The procedures followed standardized surgical protocols and techniques and were described elsewhere in detail [15-17]. The resection margin was defined as follows: R0: no residual tumor; R1: microscopic residual tumor; and R2: macroscopic residual tumor [18].

The patients were followed in the out-patient clinic at 1 month after the operative procedure and every 3 months thereafter with regular abdominal ultrasound, liver function test, and tumor markers. If there was a suspicious recurrence, computed tomography scan or magnetic resonance imaging was performed.

For patients with unresectable or recurrent disease, transarterial embolization, radio frequency ablation (RFA), percutaneous ethanol injection (PEI), chemotherapy, or combined treatments were applied.

The descriptive statistics were presented with median (range). The overall survival, disease-free survival, and survival between different histological types were determined by the Kaplan-Meier method and compared by the log-rank test. A *P* value <0.05 was considered significant. Statistical evaluation was performed using the Statistical Package for Social Sciences 18 for Windows (SPSS Inc., Chicago, IL).

## Results

There were eight male and five female (ratio: 1.6:1) patients. The median age was 48 (21 to 70) years. The demographics were listed in Table 1. Most patients (69%) presented with abdominal pain mainly in the right upper quadrant or periumbilical areas. Seven (53%) patients presented with anemia and two (15%) were with thrombocytopenia. The AFP, CEA, and CA 19-9 were within the normal reference range values except in one patient with angiosarcoma who had CA 19-9 of 337.92 U/ml.

**Table 1:** **The demographics, histology, surgical procedures, and survival of the patients**

	**Sex**	**Age**	**Preoperative biopsy**	**Pathology**	**Size (cm)**	**Operation**	**Recurrence (months)**	**Follow-up (months)**
1	M	46		Angiosarcoma	14	Right lobectomy	26.3	E 44.2
2	M	54	Angiosarcoma	Angiosarcoma	10	Right lobectomy	4.78	E 9.1
3	M	70		Angiosarcoma	2.5	Left lobectomy + PEI to S8	2.34	E 3.5
4	M	68	Sarcoma	Angiosarcoma	16	Right lobectomy	22.89	E 31.4
5	F	60	Angiosarcoma	Angiosarcoma	2.5	Nil		E 8.7
6	M	46	Angiosarcoma	Angiosarcoma	14	Nil		E 2.7
7	F	27		Embryonal sarcoma	14	Right lobectomy^b^		A 142.5
8	M	39		Leiomyosarcoma	0.6	Right + partial S1-2^a^	1.0	E 33.7
9	F	39	Neuroendocrine tumor	HEHE	2.7	Deceased donor LT^a^		A 135.9
10	F	21		Undifferentiated sarcoma	5	S4 resection + resection of solitary lung metastasis^b^		A 123.7
11	M	69	Malignant spindle cell neoplasm	Undifferentiated sarcoma	15.5	Left lobectomy^b^	3.7	E 8.2
12	M	49	Negative	Malignant mesenchymoma	4.5	Living donor LT	5.6	E 19.4
13	F	61		Undifferentiated sarcoma	16	Right lobectomy		A 65.4

*HEHE* hepatic epithelioid hemangioendothelioma, *PEI* percutaneous ethanol injection, *LT* liver transplantation, *A* alive, *E* expired, *S8* segment 8 of liver, *S1-2* segment 1 and 2 of liver, *S4* segment 4 of liver.

^a^Preoperative TAE; ^b^adjuvant chemotherapy.

Seven (53%) patients had pretreatment biopsy where six had proven malignancies and one was negative of malignancy. The positive and accurate rates of percutaneous biopsy were 85.7% and 57.1%, respectively. No complications or needle-tract tumor seeding were associated with percutaneous biopsy in this series.

Three patients had hepatitis B virus infection. One had hepatitis B and C co-infection. But no patient with angiosarcoma had viral hepatitis. All liver function tests were within normal limits except in patient 12 with hepatitis B and C co-infection where the bilirubin was 6 mg/dl.

The pathology reports showed that angiosarcoma (*n* = 6, 46%) was the most common histological type in our series. Other histological types included four (30.7%) undifferentiated sarcomas, one (7.6%) each for hepatic epithelioid hemangioendothelioma (HEHE), leiomyosarcoma, and malignant mesechymoma. Patient 8 who had leiomyosarcoma over segment 1 of the liver also had HCC in the right lobe simultaneously.

Eleven patients (84.6%) underwent surgical treatment. Among them, nine patients underwent liver resection and two underwent LT. The treatment course of each patient is described as below and is summarized in Table 1.

Patient 1 who had angiosarcoma underwent right lobectomy for a 14-cm tumor with diaphragm invasion. After 26.3 months, he had peritoneal recurrence with surgical proof. We arranged 5 cycles of systemic chemotherapy with doxorubicin, mesna, dacarbazine (DTIC), and ifosfamide and intra-artery chemotherapy once. He died because of tumor progression and liver failure.

Patient 2 had a right lobectomy for a 10-cm angiosarcoma and had multiple intrahepatic recurrence 4.8 months after resection. There was no palliative treatment.

Patient 3 had a left lobectomy for a 2.5-cm angiosarcoma and intraoperative PEI for a segment 8 liver tumor. He had an intrahepatic recurrence 2.3 months after resection. He received transarterial embolization (TAE) once. Fatal recurrent tumor rupture occurred 3.5 months after resection.

Patient 4 had a right lobectomy for a 16-cm angiosarcoma. Intra-abdominal and pulmonary metastasis happened 22.9 months after liver resection. The patient expired under supportive treatment.

Patient 5 was diagnosed with hepatic angiosarcoma with needle biopsy. She received multiple sessions of chemotherapy with adriamycin in combination with taxotere or ifosfamide. Tumor progression with rupture occurred 6 months after diagnosis and TAE was applied. She expired 8.7 months after diagnosis due to a large middle cerebral artery territory infarction.

Patient 6 was diagnosed with hepatic angiosarcoma with needle biopsy. He died 2.7 months after biopsy without further treatment.

Patient 8 who had leiomyosarcoma and HCC had TAE for huge right lobe HCC before operation. He had right lobectomy and wedge resection of segment 1 liver tumor. Recurrence occurred 1 month after resection. There were multiple sessions of TAE for recurrence. Because of poor control of recurrent tumors, we arranged two courses of chemotherapy with mitoxantrone, cisplatin, and 5-Fu. The tumor still progressed. This patient expired 33.7 months after resection.

Patient 9 who was diagnosed with neuroendocrine tumor received 6 cycles of chemotherapy with 5-Fu and leucovorin and five sessions of TAE in another hospital. She received cadaveric LT afterwards. There was no adjuvant therapy.

Patient 10 had segment 4 liver resection and combined wedge resection of undifferentiated sarcoma with solitary lung metastasis. She received 6 cycles of chemotherapy with ifosfamide, doxorubicin, dacarbazine, and mesna. The recurrence-free survival was 123.7 month at the end of this study.

Patient 11 had a left lobectomy for undifferentiated sarcoma. The pathology showed portal vein micro- and macro-vascular invasion, left hepatic vein invasion, and bile duct invasion. He received 4 cycles of chemotherapy with doxorubicin, mesna and ifosfamide, and also TAE. He died because of disease progression.

Patient 12 underwent living-related donor LT for cirrhosis and clinically diagnosed HCC. The final pathology was malignant mesenchymoma of the liver. Intrahepatic recurrence occurred 5.6 months after LT. We arranged multiple sessions of RFA and PEI. But the tumor recurred and progressed. He expired 19.4 months after LT.

Patient 13 had a right lobectomy for a 16-cm undifferentiated sarcoma. There was no adjuvant therapy. He had no recurrence till the end of this study (follow-up time: 65.4 months).

Seven patients had disease recurrence. The time of recurrence was 4.8 (1.0 ~ 26.3) months. All (4/4, 100%) angiosarcoma patients had recurrence after surgery with time to recurrence of 13.8 (2.4 ~ 26.3) months. Two (40%) patients in the undifferentiated sarcoma group had recurrence with the time of 3.7 and 5.6 months.

The resection margin status was R0 in nine patients and R1 in two patients. The 2-year survival rate of patients with R0 resection was 77.8%, but two patients with R1 resection died within 1 year (*P* = 0.006).

**Figure 1:**
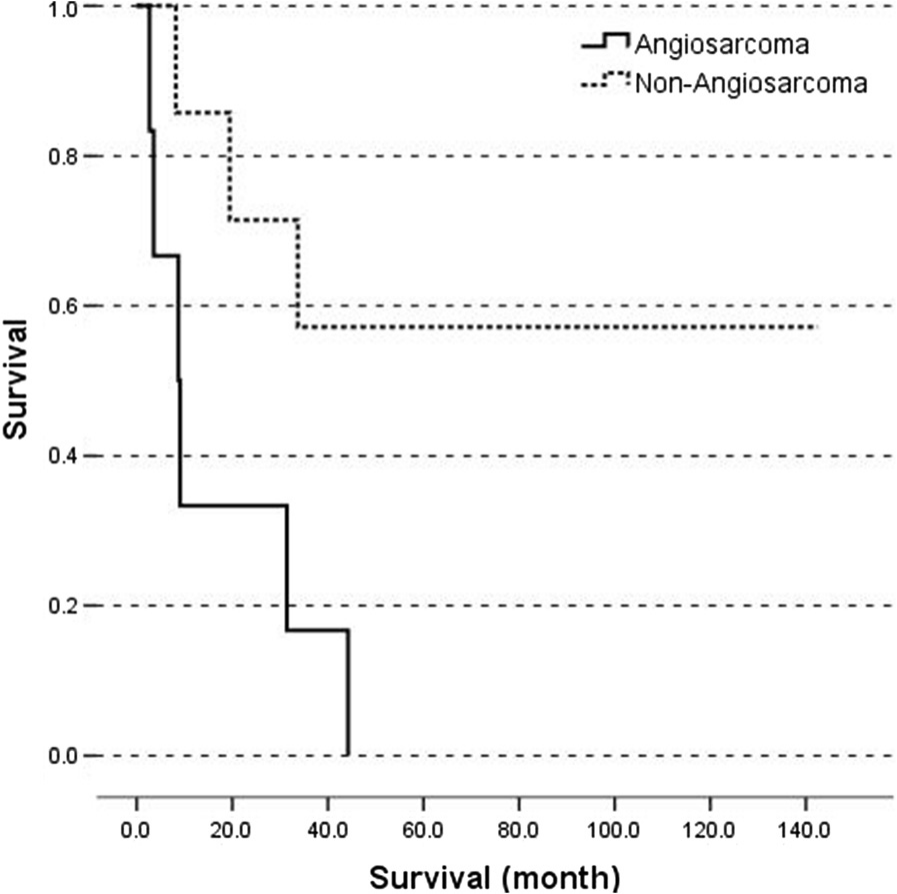
**The survival of the angiosarcoma group is significantly worse than the non-angiosarcoma group (** ***P*** **= 0.023).**

No patient lost follow-up. The median survival was 31.4 (2.7 ~ 142.5) months at the end of study. The overall 1-, 2-, and 5-year survival rates were 61.5%, 53.8%, and 30.8%, respectively. The 1-, 2-, and 5-year survival rates of surgical patients were 72.7%, 63.6%, and 36.4%, respectively. All patients with angiosarcoma expired during study time with median survival of 8.9 (2.7 ~ 44.2) months. The patients with non-angiosarcoma PHS were with median survival of 65.4 (8.2 ~ 142.5) months. The 1-, 2-, and 5-year survival rates of the angiosarcoma group were 33.3%, 16.7%, and 0%, respectively. The 1-, 2-, and 5-year survival rates of the non-angiosarcoma group were 85.7%, 71.4%, and 57.1%, respectively. The 1-, 2-, and 5-year survival rates of the undifferentiated sarcoma group (including undifferentiated sarcoma, embryonal sarcoma, and malignant mesenchymoma) were 80%, 60%, and 60%, respectively. The survival of non-angiosarcoma patients was significantly superior to those of angiosarcoma (*P* = 0.023) (Figure 1).

## Discussion

Since PHS is a relatively rare malignancy, only few series and case reports have been discussed about the management and outcome of PHS in the literature. In this study, the clinical presentations of our patients were non-specific such as abdominal pain and weight loss which were similar with other series [4,5]. Several risk factors are theorized to cause PHS but the pathogenesis still remains unclear. The recognized risk factors of angiosarcoma are exposure to thorotrast, arsenic, and vinyl chloride monomer [4]. After reviewing our patients’ personal and occupational history, no patient had contact with these materials. Unlike HCC which is highly associated with viral and alcoholic hepatitis, we found only 4 (30.7%) of the 13 patients were with viral hepatitis. Furthermore, in the angiosarcoma group, there were no patients with underlying viral hepatitis. This finding was similar to published results [19]. Besides, no specific serologic marker (such as CEA, AFP, and CA 19-9) is specific to sarcoma [6]. Hence, early detection for PHS proves to be difficult. Due to the absence of specific markers and symptoms, the reported median delay of diagnosis was 4 months and there was a 6-month delay of treatment [7]. Owing to delayed diagnosis, <20% of patients have the chance to receive radical operation [20,21].

Regarding image studies, the most favorable modalities are multiphase CT and MRI. Some of the PHSs have characteristic features but they still frequently overlap with other lesions [22]. The imaging presentation is heterogeneous within each kind of PHS or between different kinds of PHS [22,23]. Angiosarcoma might be multiple nodules, solitary huge mass, or diffusely infiltrating lesion. Of them, 17% to 27% have a chance of intraperitoneal or intratumoral hemorrhage [22-25]. Most angiosarcomas are hypoattenuating compared with normal liver. Focal areas of enhancement with bizarre shapes and central enhancement or peripheral ring-shaped enhancement is considered to be different from cavernous hemangioma. In MRI, angiosarcoma may contain areas of high-signal intensity on T1-weighted images and heterogeneous architecture on T2-weighted images [26,27]. HEHE are mostly multiple (82%). Enhanced CT usually shows marginal enhancement in the arterial phase and isodense to liver parenchymal in late phase. And there might be a halo or target sign in larger tumors [28,29]. Leiomyosarcoma in CT have been described as a large, well-defined, heterogeneous-hypodensity mass with internal and peripheral enhancement. And it can be a large hypervascular mass with hemorrhage or liquescent necrosis [30]. Undifferentiated sarcoma is usually a large, solitary and well-circumscribed mass with areas of hemorrhage, necrosis, and cystic degeneration. Tumor enhancement is usually at the delayed phase in CT. Typical MRI findings of undifferentiated sarcomas are a discordant cystic mass due to high-water content which are hypointense on T1-weighted images and have high-signal intensity on T2-weighted images. It is usually with heterogeneous enhancement in contrast study and might be more prominent on delayed-phase images [31,32]. Indeed, these PHSs have characteristic features in imaging studies, but it is still difficult to differentiate when available information is limited. Histopathology is essential for diagnosis.

The definite diagnosis of PHS should be made with tissue proof. For resectable tumor, preoperative biopsy might not be necessary. But histopathological confirmation of unresectable tumor may serve as the guide for therapies other than resection such as chemotherapy, loco-regional therapy, or even for LT. Seven (54%) patients had pretreatment percutaneous biopsy in our series. Of the pretreatment biopsies, 86% were positive for malignancy. And 57% of them were consistent with final pathology. The diagnostic rates of biopsy were similar with the results of other studies [23]. The biopsy result might not give us the accurate diagnosis. But it would help us to exclude the diagnosis of HCC. Some treatments such as LT are indicated for cirrhosis and HCC within certain criteria in order to achieve a good outcome. Our patient 12 had no tissue proof before LT and underwent LT under clinically diagnosed HCC. But explant pathology showed malignant mesenchymoma. Recurrence came early and was progressive. Definite diagnosis with tissue proof is crucial for medical treatment and transplant candidates.

As most literatures’ conclusion, complete surgical resection is the treatment of choice and provides the only possibility of long-term survival [6,7,9]. Patients with PHS did not survive more than 1 year without operation in our series. Surgical treatment showed potential benefit especially in non-angiosarcoma PHS patients; the 5-year survival rate was 57.1%. For different histological types, leiomyosarcomas are reported with poor outcome [33]. In our patient 8, he had early recurrence 1 month after resection, because the pathology showed HCC and leiomyosarcoma in different parts of the liver and we did not have tissue diagnosis after recurrence. The true histology of recurrence was not known. In previous literature, the prognosis of undifferentiated sarcoma was poor, but recent evidence shows that long-term survival is possible after radical resection with or without adjuvant chemotherapy [34]. The 5-year survival of patients with undifferentiated sarcoma was 60% in our series. And these three survivors had the follow-up time from 65.4 months to 142.5 months. Initially, our patient 10 were presented with segment 4 liver tumor and solitary lung metastasis. We arranged liver resection combined with thoracoscopic wedge resection followed by adjuvant chemotherapy. The patient survives more than 10 years without recurrence. Aggressive surgical approach should be considered seriously and might be reasonable for undifferentiated sarcoma. The vascular malignancies in PHS are HEHE and angiosarcoma. The outcome of our patient with HEHE was excellent after LT. Till the end of our study, the recurrence-free survival was 135.9 months. In contrast, the outcome of angiosarcoma was dismal. Even after potentially curative resection, none of the angiosarcoma patients survived more than 5 years and the 2-year survival rate was 16.7% only. In brief, angiosarcoma had a significantly worse outcome than non-angiosarcoma which was consistent with reports in related literature [6], and one of the important factors affecting survival was the histological type.

The radicality or extent of the operative procedure also affects the outcome significantly (*P* = 0.006). In R0 patients, 77.8% survived more than 2 years. But in patients with R1, all died within 1 year. The 5-year survival in our series (30.8%) is relative lower than in other series (37% ~ 65.2%) [1,6,7]. We believed that one of the reasons was that more patients were diagnosed with angiosarcoma in this study (46%) than in other series (16.7% ~ 22.7%).

The indication of PHS undergoing LT depends on the type of histopathology. Due to early recurrence and poor outcome, angiosarcoma is considered as an absolute contraindication [35]. The most favorable type of PHS for LT is HEHE. Gores et al. reported that the 1-, 5-, and 10-year patient survival rates from HEHE after LT were 93%, 83%, and 72%, respectively [36], which are similar or even better than the results of LT for other diseases. Our patient 9 with HEHE underwent LT and is currently alive without recurrence to date for more than 11 years. In contrast, our patient 12 who had malignant mesenchymoma underwent LT and early recurrence occurred. Despite multiple sessions of locoregional therapy, he expired 19.4 months after LT. Regarding histological types other than HEHE, the indication for LT is still controversial [35].

## Conclusions

In conclusion, despite the limited number of patients in this series, we found that angiosarcoma is associated with a much worse outcome than other types. We proposed that PHS could be separated into ‘angiosarcoma’ and ‘non-angiosarcoma.’ Radical surgical resection offers the only opportunity to achieve long-term survival. Early diagnosis is critical to increase the possibility of resection, but it is difficult to make an early detection. Unresectable HEHE is an indication for LT where long-term survival may be expected.

## Abbreviations

AFP: alpha-fetoprotein
CA 19-9: carbohydrate antigen 19-9
CEA: carcinoembryonic antigen
CT: computed tomography
HCC: hepatocellular carcinoma
HEHE: hepatic epithelioid hemangioendothelioma
LT: liver transplantation
MRI: magnetic resonance imaging
PHS: primary hepatic sarcoma
